# Long-term effectiveness and tolerability of dolutegravir/lamivudine in treatment-naive people with HIV: an analysis of a multicentre cohort at 96 weeks

**DOI:** 10.1093/jac/dkae456

**Published:** 2024-12-23

**Authors:** Inés Suárez-García, Belén Alejos, Cristina Moreno, Juan Martín Torres, Mar Masiá, Lucio J García-Fraile, Melchor Riera, David Dalmau, Rafael Rodríguez-Rosado, Roberto Muga, Santiago Moreno, Inma Jarrín, the CoRIS cohort, Santiago Moreno, Inma Jarrín, David Dalmau, M Luisa Navarro, M Isabel González, Federico Garcia, Eva Poveda, Jose Antonio Iribarren, Félix Gutiérrez, Rafael Rubio, Francesc Vidal, Juan Berenguer, Juan González, M Ángeles Muñoz-Fernández, Inmaculada Jarrín, Cristina Moreno, Marta Rava, Rebeca Izquierdo, Cristina Marco, Teresa Gómez-García, M Ángeles Muñoz-Fernández, Roxana Juárez, Joaquín Portilla, Irene Portilla, Esperanza Merino, Gema García, Iván Agea, José Sánchez-Payá, Juan Carlos Rodríguez, Livia Giner, Sergio Reus, Vicente Boix, Diego Torrus, Verónica Pérez, Julia Portilla, Héctor Pinargote, María Remedios Alemán, Ana López Lirola, Dácil García, Felicitas Díaz-Flores, M Mar Alonso, Ricardo Pelazas, María Inmaculada Hernández, Lucia Romero, Abraham Bethencourt, Daniel Rodríguez, Víctor Asensi, María Eugenia Rivas-Carmenado, Rebeca Cabo Magadan, Javier Díaz-Arias, Federico Pulido, Rafael Rubio, Otilia Bisbal, M Asunción Hernando, David Rial, María de Lagarde, Adriana Pinto, Laura Bermejo, Mireia Santacreu, Roser Navarro, Juan Martín Torres, José Antonio Iribarren, M José Aramburu, Xabier Camino, Miguel Ángel Goenaga, M Jesús Bustinduy, Harkaitz Azkune, Maialen Ibarguren, Xabier Kortajarena, Ignacio Álvarez-Rodriguez, Leire Gil, Francisco Carmona-Torre, Ana Bayona Carlos, Maialen Lekuona Sanz, Félix Gutiérrez, Catalina Robledano, Mar Masiá, Sergio Padilla, Araceli Adsuar, Rafael Pascual, Marta Fernández, Antonio Galiana, José Alberto García, Xavier Barber, Javier García Abellán, Guillermo Telenti, Lucía Guillén, Ángela Botella, Paula Mascarell, Mar Carvajal, Alba de la Rica, Carolina Ding, Lidia García-Sánchez, Nuria Ena, Leandro López, Jennifer Vallejo, Nieves Gonzalo-Jiménez, Montserrat Ruiz, Christian Ledesma, Santiago López, María Espinosa, Ana Quiles, María Andreo, Roberto Muga, Arantza Sanvisens, Daniel Fuster, Juan Carlos López Bernaldo de Quirós, Isabel Gutiérrez, Juan Berenguer, Margarita Ramírez, Paloma Gijón, Teresa Aldamiz-Echevarría, Francisco Tejerina, Cristina Diez, Leire Pérez, Chiara Fanciulli, Saray Corral, Joaquín Peraire, Anna Rull, Anna Martí, Consuelo Viladés, Beatriz Villar, Lluïsa Guillem, Montserrat Olona, Graciano García-Pardo, Frederic Gómez-Bertomeu, Verónica Alba, Silvia Chafino, Alba Sánchez, Marta Montero, María Tasias, Eva Calabuig, Miguel Salavert, Juan Fernández, Rosa Blanes, Juan González-García, Ana Delgado-Hierro, José Ramón Arribas, Víctor Arribas, José Ignacio Bernardino, Carmen Busca, Joanna Cano-Smith, Julen Cadiñanos, Juan Miguel Castro, Luis Escosa, Iker Falces, Pedro Herranz, Víctor Hontañón, Alicia González-Baeza, M Luz Martín-Carbonero, Mario Mayoral, Rafael Micán, Rosa de Miguel, Rocío Montejano, Mª Luisa Montes, Luis Ramos-Ruperto, Berta Rodés, Talía Sainz, Elena Sendagorta, Eulalia Valencia, M del Mar Arcos, Alejandro de Gea Grela, Carlos Oñoro López, José Ramón Blanco, Laura Pérez-Martínez, José Antonio Oteo, Valvanera Ibarra, Luis Metola, Mercedes Sanz, Rosa Martínez, Desiré Gil, Álvaro Cecilio, Ruth Caballero, María Aranzazu Caudevilla, David Dalmau, Marina Martinez, Angels Jaén, Mireia Cairó, Javier Martinez-Lacasa, Roser Font, Laura Gisbert, María Rivero, Maider Goikoetxea, María Gracia, Carlos Ibero, Estela Moreno, Jesús Repáraz, Fernando Baigorria, Gemma Navarro, Manel Cervantes Garcia, Sonia Calzado Isbert, Marta Navarro Vilasaro, Ignacio de los Santos, Alejandro de los Santos, Lucio García-Fraile, Enrique Martín, Ildefonso Sánchez-Cerrillo, Marta Calvet, Ana Barrios, Azucena Bautista, Carmen Sáez, Marianela Ciudad, Ángela Gutiérrez, María Aguilera García, Santiago Moreno, Santos del Campo, José Luis Casado, Fernando Dronda, Ana Moreno, M Jesús Pérez, Sergio Serrano-Villar, Mª Jesús Vivancos, Javier Martínez-Sanz, Alejandro Vallejo, Matilde Sánchez-Conde, José Antonio Pérez-Molina, José Manuel Hermida, Erick De La Torre Tarazona, Elena Moreno, Laura Martín Pedraza, Claudio Díaz García, Jorge Díaz, Alejandro García, Raquel Ron, Enrique Bernal, Antonia Alcaraz, Joaquín Bravo, Ángeles Muñoz, Cristina Tomás, Eva Oliver, David Selva, Eva García, Román González, Elena Guijarro, Rodrigo Martínez, María Dolores Hernández, Federico García, Clara Martínez, Leopoldo Muñoz Medina, Marta Álvarez, Natalia Chueca, David Vinuesa, Adolfo de Salazar, Ana Fuentes, Emilio Guirao, Laura Viñuela, Andrés Ruiz-Sancho, Francisco Anguita, Naya Faro, José Peregrina, Lucia Chaves, Marta Illescas, Valme Sánchez, Jorge Del Romero, Montserrat Raposo, Carmen Rodríguez, Teresa Puerta, Juan Carlos Carrió, Mar Vera, Juan Ballesteros, Oskar Ayerdi, Begoña Baza, Eva Orviz, Antonio Antela, Elena Losada, Melchor Riera, María Peñaranda, M Angels Ribas, Antoni A Campins, Mercedes Garcia-Gazalla, Francisco J Fanjul, Javier Murillas, Francisco Homar, Helem H Vilchez, Luisa Martin, Antoni Payeras, Jesús Santos, María López, Cristina Gómez, Isabel Viciana, Rosario Palacios, Luis Fernando López-Cortés, Nuria Espinosa, Cristina Roca, Silvia Llaves, Juan Manuel Tiraboschi, Arkaitz Imaz, María Saumoy, Adrián Curran, Vicenç Falcó, Jordi Navarro, Joaquin Burgos, Paula Suanzes, Jorge García, Vicente Descalzo, Patricia Álvarez, Bibiana Planas, Marta Sanchíz, Lucía Rodríguez, Arnau Monforte, Paola Vidovic, Julián Olalla, Javier Pérez, Alfonso del Arco, Javier de la Torre, José Luis Prada, Onofre Juan Martínez, Lorena Martinez, Francisco Jesús Vera, Josefina García, Begoña Alcaraz, Antonio Jesús Sánchez Guirao, Álvaro Mena, Berta Pernas, Pilar Vázquez, Soledad López, Brais Castelo, Sofía Ibarra, Guillermo García, Josu Mirena, Oscar Luis Ferrero, Josefina López, Mireia de la Peña, Miriam López, Iñigo López, Itxaso Lombide, Víctor Polo, Joana de Miguel, Beatriz Ruiz Estevez, Maite Ganchegui Aguirre, María Jesús Barberá Gracia, Carlos Galera, Marian Fernández, Helena Albendin, Antonia Castillo, Asunción Iborra, Antonio Moreno, M Angustias Merlos, Inmaculada Chiclano, Concha Amador, Francisco Pasquau, Concepción Gil, José Tomás Algado, Inés Suarez-García, Eduardo Malmierca, Patricia González-Ruano, M Pilar Ruiz, José Francisco Pascual, Luz Balsalobre, Ángela Somodevilla, María de la Villa López, Mohamed Omar, Carmen Herrero, Miguel Alberto de Zárraga, Desirée Pérez, Vicente Estrada, Noemí Cabello, M José Núñez, Iñigo Sagastagoitia, Reynaldo Homen, Ana Muñoz, Inés Armenteros Yeguas, Miguel Górgolas, Alfonso Cabello, Beatriz Álvarez, Laura Prieto, Aws Al-Hayani, Irene Carrillo, José Sanz, Alberto Arranz, Cristina Hernández, María Novella, M José Galindo, Sandra Pérez Gómez, Ana Ferrer, Antonio Rivero Román, Inma Ruíz, Antonio Rivero Juárez, Pedro López, Isabel Machuca, Mario Frias, Ángela Camacho, Ignacio Pérez, Diana Corona, Javier Manuel Caballero, Rafael Rodríguez-Rosado Martinez-Echevarría, Rafael Torres, Juan Macías Sánchez, Pilar Rincón, Luis Miguel Real, Anais Corma, Alejandro González-Serna, Eva Poveda, Alexandre Pérez, Luis Morano, Celia Miralles, Antonio Ocampo, Guillermo Pousada, María Gallego, Jacobo Alonso, Inés Martínez, Carlos Dueñas, Sara Gutiérrez, Marta de la Fuente López, Cristina Novoa, Xjoylin Egües, Pablo Telleria, Carlos Güerri Fernández, Claudia Navarro Valls, Juan Du, Agustin Marcos Blanco, Itziar Arrieta Aldea, Esperanza Cañas Ruan, Cecilia Canepa, Natalia García Giralt

**Affiliations:** Infectious Diseases Group, Department of Internal Medicine, Hospital Universitario Infanta Sofia, FIIB HUIS HUHEN, Madrid, Spain; CIBER de Enfermedades Infecciosas (CIBERINFEC), Carlos III Health Institute, Madrid, Spain; Clinical Department, Facultad de Medicina, Salud y Deporte, Universidad Europea de Madrid, Madrid, Spain; Independent Researcher, Madrid, Spain; CIBER de Enfermedades Infecciosas (CIBERINFEC), Carlos III Health Institute, Madrid, Spain; National Centre for Epidemiology, Carlos III Health Institute, Madrid, Spain; HIV Unit, Department of Internal Medicine, Hospital Universitario 12 de Octubre, Madrid, Spain; CIBER de Enfermedades Infecciosas (CIBERINFEC), Carlos III Health Institute, Madrid, Spain; Infectious Diseases Section, Department of Internal Medicine, Hospital General Universitario de Elche, Alicante, Spain; Department of Clinical Medicine, Universidad Miguel Hernández de Elche, Alicante, Spain; CIBER de Enfermedades Infecciosas (CIBERINFEC), Carlos III Health Institute, Madrid, Spain; Infectious Diseases Unit, Department of Internal Medicine, Hospital Universitario de La Princesa, Madrid, Spain; CIBER de Enfermedades Infecciosas (CIBERINFEC), Carlos III Health Institute, Madrid, Spain; Department of Internal Medicine, Section of Infectious Diseases, Hospital Universitario Son Espases-IDISBA, Palma, Spain; HIV/STI/PrEP Unit, Infectious Disease Department, Hospital Universitari Mutua Terrassa, Terrassa, Spain; Department of Medicine, Universitat de Barcelona, Barcelona, Spain; Department of Internal Medicine, Hospital Universitario Severo Ochoa, Madrid, Spain; Department of Internal Medicine, Hospital Universitari Germans Trias i Pujol, Badalona, Spain; Department of Medicine, Universitat Autònoma de Barcelona, Barcelona, Spain; CIBER de Enfermedades Infecciosas (CIBERINFEC), Carlos III Health Institute, Madrid, Spain; Department of Infectious Diseases, Ramón y Cajal University Hospital, IRYCIS, Madrid, Spain; Department of Medicine, Universidad de Alcalá, Madrid, Spain; CIBER de Enfermedades Infecciosas (CIBERINFEC), Carlos III Health Institute, Madrid, Spain; National Centre for Epidemiology, Carlos III Health Institute, Madrid, Spain

## Abstract

**Objectives:**

To evaluate the long-term effectiveness, persistence and tolerability of dolutegravir (DTG)/lamivudine (3TC), compared with the most frequently prescribed first-line treatment regimens, among antiretroviral-naive people with HIV from CoRIS, a multicentre cohort in Spain, in 2018–23.

**Methods:**

We used multivariable regression models to compare viral suppression (VS) (HIV RNA viral load <50 copies/mL), change in CD4 cell counts, persistence and treatment discontinuations due to adverse events (AEs), at 96 (±24) weeks after treatment initiation.

**Results:**

Of 2359 participants, DTG/3TC was prescribed in 472 (20.0%), bictegravir/tenofovir alafenamide (TAF)/emtricitabine (FTC) in 1134 (48.1%), DTG + tenofovir disoproxil fumarate/FTC in 300 (12.7%), DTG/abacavir/3TC in 273 (11.6%) and darunavir/cobicistat/TAF/FTC in 180 (7.6%). At 96 weeks from treatment initiation, 94.0% of participants initiating with DTG/3TC achieved VS, and the mean increase in CD4 cell counts was 295.5 cells/μL (95% CI: 269.9–321.1). During the first 96 weeks after DTG/3TC initiation, 9.8% and 1.3% discontinued their initial regimen, overall and due to AEs, respectively. In multivariable analyses, we did not find significant differences in VS or increase in CD4 cell counts among participants initiating with DTG/3TC compared with other regimens. Initiating ART with a regimen other than DTG/3TC was associated with a higher risk of treatment discontinuation, overall and due to AEs.

**Conclusions:**

Among treatment-naive people with HIV from this large multicentre cohort, DTG/3TC had similar effectiveness and better persistence and tolerability than those of the most frequently prescribed first-line regimens at 96 weeks.

## Introduction

Dolutegravir (DTG)/lamivudine (3TC) has shown high efficacy as a first-line antiretroviral treatment (ART) for people with HIV (PWH) in clinical trials^1,2^ and is now recommended in the main clinical practice guidelines for treatment-naive and virally suppressed PWH.^3–5^ The use of DTG/3TC in clinical practice has allowed an increasing number of cohort studies that have confirmed its effectiveness in diverse real-world settings.^6^ Evidence from cohort studies is important, as they usually include participants that are more representative of the general population than those from clinical trials, and they take place in settings that better relate to day-to-day clinical practice.^7^

As PWH need life-long ART, it is important to assess its long-term effectiveness and durability. Results from the GEMINI clinical trials have confirmed the efficacy, safety and high barrier to resistance of DTG/3TC at 96 and 144 weeks in treatment-naive participants.^8,9^ However, there is very little evidence on long-term effectiveness of DTG/3TC from cohort studies. Specifically, in treatment-naive participants, only two cohorts have assessed its effectiveness at specific timepoints beyond 48 weeks: REDOLA, including 185 participants in Spain,^10^ and URBAN, including 31 participants in Germany.^11^ Both were descriptive studies, and neither of them compared the effectiveness of DTG/3TC with other first-line treatments.

We have previously published the treatment outcomes of treatment-naive and virally suppressed participants at 48 weeks after initiation of DTG/3TC from CoRIS, a large multicentre cohort of PWH in Spain.^12^ The aims of this study were to assess the effectiveness, tolerability and persistence of DTG/3TC at 96 weeks among treatment-naive participants in the CoRIS cohort, compared with other first-line ARTs, and to assess its effectiveness and tolerability in specific subgroups that are underrepresented in clinical trials and might be at risk of suboptimal response to ART in the long term.

## Materials and methods

### Study design

CoRIS is an open, multicentre and prospective cohort of adults with HIV who are naive to ART at study entry. Participants in CoRIS were seen for the first time in specialist HIV clinics, recruited from 1 January 2004 in any of the 48 participating centres from 14 of 17 autonomous regions in Spain and followed up until 30 November 2023, the administrative censoring date for these analyses.

Briefly, CoRIS collects a minimum dataset as provided for in the cohort protocol, which includes baseline and follow-up sociodemographic, immunological and clinical data including data on antiretroviral medications with start and stop dates and reasons for drug discontinuation. Data are highly standardized and submitted to periodic quality control procedures. Individuals are followed up periodically in accordance with routine clinical practice.^13^

### Study population

For this study, we included ART-naive individuals from the CoRIS cohort, aged ≥ 18 years, who started ART with DTG/3TC or other first-line regimens between 1 August 2018 and 30 November 2021 to ensure that all participants could be followed for at least 96 weeks. For all the analyses, we excluded individuals (i) who started ART in the context of a clinical trial or (ii) with no follow-up after ART initiation. Only regimens prescribed in >5% of individuals were considered.

### Outcomes

The primary outcomes were as follows: (i) viral suppression (VS), defined as an HIV RNA viral load (VL) <50 copies/mL at 96 (±24) weeks after initiation of ART, and (ii) virological failure (VF) after VS, defined as two consecutive HIV RNA VL levels >50 copies/mL or one >1000 copies/mL after VS and prior to 96 weeks after initiation.

The secondary outcomes included: (i) immunological response (IR), defined as the change in CD4 cell counts at 96 (±24) weeks after ART initiation, (ii) persistence, defined as time-to-treatment discontinuation (i.e. stopping or changing any component of the regimen) during the first 96 weeks after initiation of ART and reason for discontinuation and (iii) incidence of treatment discontinuations due to adverse events (AEs) over 96 weeks after initiation and description of the AE.

Reasons for treatment discontinuation were classified as treatment failure, AE, simplification, drug interaction, patient’s wish/decision, pregnancy, enrolment in a clinical trial, cost reduction, toxicity prevention, other and unknown. In turn, AEs were classified as neuropsychiatric (headache, dizziness, fatigue, insomnia, sleep disturbance, anxiety/depression, emotional instability), renal, gastrointestinal (nauseas/vomiting, diarrhoea, abdominal pain), skin, liver, weight gain, other and unknown.

For the analyses of VS and IR at 96 weeks after ART initiation, only cases with available data within the assessment window were included; when more than one measurement was available within that window, we used the last available one. For the analyses of VF after VS, only cases with at least one measurement available after VS were included. We performed both ITT and on-treatment (OT) analyses. For ITT analysis, outcomes were analysed by initial regimen and later changes in the regimen were ignored; therefore, once a participant started a regimen, he/she was assumed to remain on it. For OT analysis, participants who changed their initial regimen before 96 weeks were excluded.

### Statistical analysis

Descriptive analyses were carried out using frequency tables for categorical variables and median and IQR for continuous variables. Differences in sociodemographic and clinical characteristics at ART initiation were assessed with the non-parametric Kruskal–Wallis test for continuous variables and the χ^2^ test for independence for categorical variables.

We used logistic and linear regression, respectively, to assess differences by first-line regimen in the proportion of individuals who achieved VS and the mean change in CD4 cell count at 96 (±24) weeks after ART initiation. Additionally, among individuals who achieved VS any time within the first 96 weeks, we calculated the proportion who experienced VF prior to the assessment timepoint.

To describe persistence, we used the multiple decrement method to compute the cumulative incidence curve of treatment discontinuation, considering deaths before the discontinuation as competing events, and used proportional hazards models on the sub-distribution hazard to evaluate differences according to the initial regimen. The incidence rate of treatment discontinuations due to AE per 1000 persons-year was calculated, and Poisson’s regression was used to assess differences by first-line regimen. For both analyses of persistence and incidence of treatment discontinuations due to AEs, follow-up started at ART initiation and ended at date of any treatment discontinuation or discontinuation due to AE, respectively, death, last study contact or after 96 weeks, whichever arose first.

In subgroup analyses, we specifically assessed VS and the incidence of treatment discontinuations due to AE in participants who started ART with (i) CD4 < 200 cells/μL, (ii) HIV RNA VL >100 000 copies/mL, (iii) HIV RNA VL >500 000 copies/mL, (iv) in those who started ART within 7 days from cohort enrolment, (v) in women and (vi) in those aged ≥50 years.

Multivariable models were adjusted for the following potential confounders: sex (male, female), age at ART initiation (<30, 30–49, ≥50 years), transmission category (sex between men, sex between men and women, other/unknown), educational level (no education/compulsory education, secondary/university education, other/unknown), country of origin (Spain, no Spain, unknown), CD4 cell count (<200, 200–500, >500 cells/μL, unknown) and VL (≤100,000, >100 000 copies/mL, unknown) within 6 months previous to ART initiation, HCV antibodies (no, yes, unknown), HBV surface antigen (no, yes, unknown) and previous AIDS diagnosis at ART initiation (no, yes). To adjust for clustering of participants within centres, robust methods were used to estimate standard errors. Wald’s tests were used to derive *P* values.

All statistical analyses were performed using Stata software (version 18.0; Stata Corporation, College Station, TX, USA).

### Ethics approval and informed consent

CoRIS cohort was approved by the Clinical Research Ethics Committee of the Gregorio Marañón General University Hospital. All participants agreed to participate in CoRIS by signing an informed consent form. This study was approved by the Ethics Committee of the Instituto de Salud Carlos III, Madrid, Spain (CEI PI 86_2020-v2).

## Results

During the study period, 3121 PWH aged ≥18 years started ART. Of those, we excluded 326 (10.4%) who initiated ART in the context of a clinical trial, 18 (0.6%) with no follow-up after ART initiation and 418 (13.4%) who initiated a treatment prescribed in <5% of individuals. Finally, 2359 PWH were included; their sociodemographic and clinical characteristics at ART initiation are given in Table S1 (available as Supplementary data at *JAC* Online).

DTG/3TC was prescribed in 472 (20.0%) PWH, bictegravir (BIC)/tenofovir alafenamide (TAF)/emtricitabine (FTC) in 1134 (48.1%), DTG + FTC/tenofovir disoproxil fumarate (TDF) in 300 (12.7%), DTG/abacavir (ABC)/3TC in 273 (11.6%) and darunavir (DRV)/cobicistat (COBI)/TAF/FTC in 180 (7.6%). DTG/3TC was available as a single treatment regimen in Spain in January 2020, and therefore, in all centres, it was prescribed as two separate pills until January 2020 and as a single pill thereafter. The proportion of PWH who started DTG/3TC increased gradually from 4.4% in 2018 to 24.8% in 2021. PWH who initiated ART with DTG/3TC were more frequently younger men, had contracted HIV through sex between men and had secondary or university studies. Additionally, they often started ART with a higher CD4 cell count and a VL ≤100.000 copies/mL and without a previous AIDS diagnosis (Table S1). A total of 1336 (56.6%) PWH started treatment in the first week after cohort enrolment; among PWH starting DTG/3TC, 214/472 (45.3%) started treatment in the first week.

### VS, IR and VF

Overall, in ITT analyses, VS and IR at 96 weeks after ART initiation were evaluated in 1798 (76.2%) and 1687 (71.5%) PWH, respectively. Reasons for exclusion in these analyses are detailed in Table S2. Briefly, we failed to find significant differences in the sociodemographic and clinical characteristics of participants included and excluded except for a slightly higher percentage of participants with CD4 cell count <200 cells/μL and VL >100 000 copies/mL among those included (data not shown).

At 96 weeks from ART initiation, 94.0% of PWH initiating with DTG/3TC achieved VS, and the mean increase in CD4 cell counts was 295.5 cells/μL (95% CI: 269.9–321.1). In multivariable analyses, we did not find significant differences in VS and IR among participants initiating with DTG/3TC or other regimens (Table 1). Similar results were observed in the OT analyses (Table 1).

**Table 1.** VS, IR and VF at 96 weeks from ART initiation according to first-line antiretroviral regimen, CoRIS cohort, 2018–23

	VS		IR		VF
	*N*/*N* with data (%)	Adjusted OR (95% CI)^a^	Mean increase in CD4 cell count (95% CI)	Adjusted mean difference in CD4 cell count increase (95% CI)^a^	*N*/*N* with data (%)
ITT analyses					
DTG/3TC	328/349 (94.0)	Ref.	295.5 (269.9; 321.1)	Ref.	6/386 (1.6)
BIC/FTC/TAF	791/881 (89.8)	0.87 (0.48; 1.57)	315.6 (299.1; 332.1)	10.82 (−22.93; 44.56)	63/972 (6.5)
DTG/3TC/ABC	192/207 (92.8)	1.22 (0.52; 2.82)	309.3 (276.8; 341.7)	13.67 (−30.09; 57.43)	17/235 (7.2)
DRV/COBI/FTC/TAF	123/135 (91.1)	1.12 (0.50; 2.52)	294.5 (248.7; 340.2)	−2.57 (−69.88; 64.73)	8/141 (5.7)
DTG + FTC/TDF	199/226 (88.1)	0.93 (0.45; 1.92)	362.6 (326.0; 399.1)	43.09 (7.35; 78.82)	13/241 (5.4)
OT analyses					
DTG/3TC	302/318 (95.0)	Ref.	300.6 (274.5; 326.6)	Ref.	4/354 (1.1)
BIC/FTC/TAF	703/771 (91.2)	0.76 (0.36; 1.60)	318.0 (300.2; 335.7)	13.34 (−24.87; 51.55)	42/852 (4.9)
DTG/3TC/ABC	137/145 (94.5)	1.28 (0.43; 3.79)	314.6 (274.9; 354.3)	14.29 (−37.67; 66.24)	13/163 (8.0)
DRV/COBI/FTC/TAF	87/91 (95.6)	1.67 (0.45; 6.13)	281.9 (222.0; 341.9)	−9.87 (−85.45; 65.70)	3/91 (3.3)
DTG + FTC/TDF	55/59 (93.2)	1.54 (0.43; 5.51)	309.8 (240.2; 379.4)	−14.16 (−66.46; 38.13)	3/64 (4.7)

^a^Adjusted for sex, age at ART initiation, transmission category, educational level, country of origin, CD4 cell count and VL within 6 months previous to ART initiation, presence of HCV antibodies, presence of HBV surface antigen and previous AIDS diagnosis at ART initiation.

Of the PWH who started DTG/3TC and achieved VS anytime within the following 96 weeks, 6 (1.6%) subsequently experienced VF. They are described in detail in Tables S3A and S3B. Two of them had stopped taking their treatment and were not receiving any ART at the time of failure. Of the remaining four PWH on treatment at VF, three were still receiving DTG/3TC and one was receiving DTG/ABC/3TC. After VF, the two PWH who were not receiving treatment reinitiated DTG/3TC and the four PWH who were on treatment did not change their antiretroviral regimen (three of them continued with DTG/3TC and one continued with DTG/ABC/3TC). Among those PWH with VF who had resistance testing available, no *de novo* resistance mutations were documented.

### Persistence and tolerability

**Figure 1.**
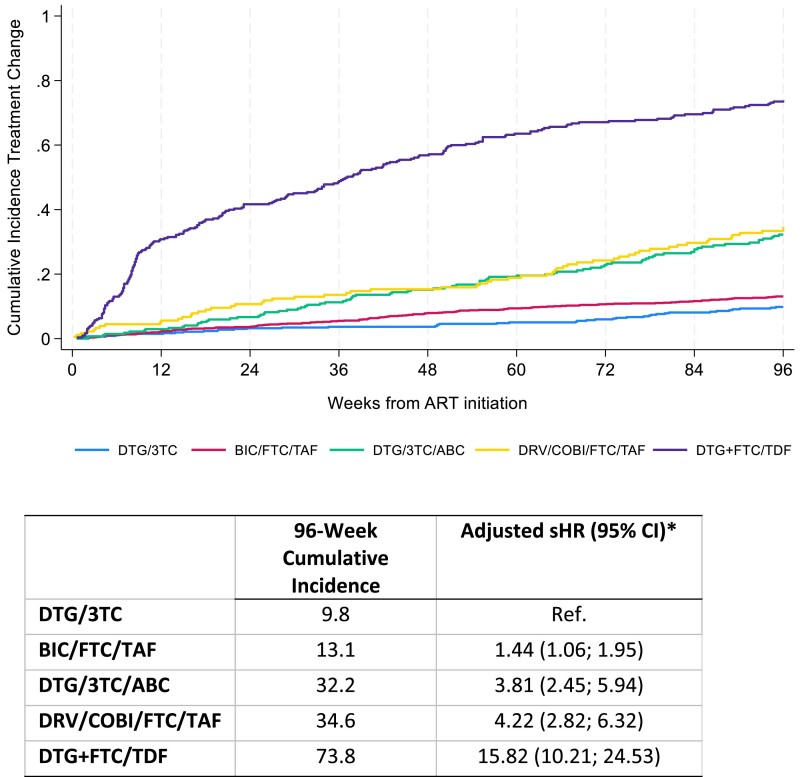
Cumulative incidence curve of treatment discontinuation during the first 96 weeks after ART initiation according to first-line antiretroviral regimen, CoRIS cohort, 2018–23. sHR, sub-distribution hazard ratio. *Adjusted for sex, age at ART initiation, transmission category, educational level, country of origin, CD4 cell count and VL within 6 months previous to ART initiation, presence of HCV antibodies, presence of HBV surface antigen and previous AIDS diagnosis at ART initiation.

During the first 96 weeks of ART initiation, 9.8% of PWH who started DTG/3TC discontinued their initial regimen. The percentage of discontinuations varied between 13.1% and 73.8% among the other regimens analysed. In multivariable analysis, initiating ART with a regimen other than DTG/3TC was associated with a higher risk of treatment discontinuation (Figure 1). Simplification was the most frequent reason for treatment discontinuation in all regimens except DTG/3TC. A detailed description of reasons for treatment discontinuation and substitution regimens is given in Table 2.

**Table 2.** Reasons for treatment discontinuation during the first 96 weeks after ART initiation and substitution regimen, according to first-line regimen, CoRIS cohort, 2018–23

	DTG/3TC	BIC/FTC/TAF	DTG/3TC/ABC	DRV/COBI/FTC/TAF	DTG + FTC/TDF
	*n* = 472	*n* = 1134	*n* = 273	*n* = 180	*n* = 300
No. of treatment discontinuations	43	140	82	60	216
Reason for treatment discontinuation [*N* (%)]					
Treatment failure	5 (1.1)	13 (1.1)	2 (0.7)	3 (1.7)	3 (1.0)
AE	6 (1.3)	31 (2.7)	16 (5.9)	10 (5.6)	36 (12.0)
Simplification	4 (0.8)	27 (2.4)	48 (17.6)	16 (8.9)	136 (45.3)
Drug interaction	1 (0.2)	7 (0.6)	1 (0.4)	9 (5.0)	3 (1.0)
Patient’s wish/decision	9 (1.9)	9 (0.8)	3 (1.1)	2 (1.1)	2 (0.7)
Pregnancy	1 (0.2)	11 (1.0)	0	3 (1.7)	1 (0.3)
Enrolment in a clinical trial	4 (0.8)	8 (0.7)	1 (0.4)	3 (1.7)	1 (0.3)
Cost reduction	0 (0.0)	1 (0.1)	0	0	1 (0.3)
Toxicity prevention	2 (0.4)	11 (1.0)	2 (0.7)	2 (1.1)	15 (5.0)
Other	8 (1.7)	18 (1.6)	7 (2.6)	8 (4.4)	15 (5.0)
Unknown	3 (0.6)	4 (0.4)	2 (0.7)	4 (2.2)	3 (1.0)
Most frequent substitution regimens (%)	BIC/FTC/TAF (27.9)	DTG/3TC (24.3)	DTG/3TC (64.6)	BIC/FTC/TAF (38.3)	DTG/3TC (45.8)
	DRV/COBI/FTC/TAF (16.3)	DRV/COBI/FTC/TAF (17.1)	BIC/FTC/TAF (13.4)	DTG/3TC (16.7)	DTG/3TC/ABC (27.8)
	CAB + RPV (11.6)	DTG/3TC/ABC (7.1)	DRV/COBI/FTC/TAF (6.1)	DTG/RPV (8.3)	BIC/FTC/TAF (11.6)

Treatment discontinuations due to AEs are given in Table 3. The percentage of discontinuations of DTG/3TC due to AEs was 1.3%; the rate of treatment discontinuations was lower for DTG/3TC than for all the other regimens, even after adjusting for other risk factors (Table 3). The most common AE leading to discontinuation was neuropsychiatric toxicity for all regimens except DRV/COBI/FTC/TAF and DTG + FTC/TDF (Table 3).

**Table 3.** Treatment discontinuation due to AEs during the first 96 weeks after ART initiation, type of AE and substitution regimen, according to first-line antiretroviral regimen, CoRIS cohort, 2018–23

	DTG/3TC	BIC/FTC/TAF	DTG/3TC/ABC	DRV/COBI/FTC/TAF	DTG + FTC/TDF
	*n* = 472	*n* = 1134	*n* = 273	*n* = 180	*n* = 300
Treatment discontinuations due to AEs [N (%)]	6 (1.3)	31 (2.7)	16 (5.9)	10 (5.6)	36 (12.0)
Incidence rate (95% CI) × 100 py	0.8 (0.3; 1.7)	1.7 (1.2; 2.5)	4.1 (2.5; 6.7)	3.8 (2.1; 7.1)	14.1 (10.2; 19.5)
Adjusted RR (95% CI)^a^	Ref.	2.06 (1.02; 4.18)	5.55 (2.09; 14.75)	5.39 (2.38; 12.24)	17.07 (8.76; 33.30)
Type of AE [*n* (%)]					
Gastrointestinal	1 (0.2)	5 (0.4)	3 (1.1)	4 (2.2)	2 (0.7)
Liver	0	1 (0.1)	1 (0.4)	0	0
Neuropsychiatric	5 (1.1)	11 (1.0)	5 (1.8)	0	7 (2.3)
Renal	0	2 (0.2)	0	0	21 (7.0)
Skin	0	6 (0.5)	1 (0.4)	5 (2.8%)	4 (1.3)
Weight Gain	0	2 (0.2)	1 (0.4)	0	0
Other	0	3 (0.3)	5 (1.8)	1 (0.6%)	2 (0.7)
Unknown	0	1 (0.1)	0	0	0
Most frequent substitution regimens (%)	RPV + FTC/TAF (33.3)	DRV/COBI/FTC/TAF (25.8)	DTG/3TC (25.0)	BIC/FTC/TAF (40.0)	DTG/3TC/ABC (27.8)
	BIC/FTC/TAF (16.7)	DTG/3TC (22.6)	RAL + 3TC/ABC (18.7)	DTG/3TC (20.0)	BIC/FTC/TAF (19.4)
	DRV/COBI/FTC/TAF (16.7)	DTG/3TC/ABC (9.7)	EVG/COBI/FTC/TAF (18.7)	DTG/3TC/ABC (10.0)	DTG/3TC (19.4)

RR, rate ratio; py, persons-year; RAL + 3TC/ABC, raltegravir + lamivudine/abacavir; EVG/COBI/FTC/TAF, elvitegravir/cobicistat/emtricitabine/tenofovir alafenamide.

^a^Adjusted for sex, age at ART initiation, transmission category, educational level, country of origin, CD4 cell count and VL within 6 months previous to ART initiation, presence of HCV antibodies, presence of HBV surface antigen and previous AIDS diagnosis at ART initiation.

### Effectiveness and tolerability in specific subgroups

Table S4 summarizes the results of VS and treatment discontinuations due to AEs at 96 weeks after ART initiation according to first-line regimens in specific subgroups. For the subgroup who started treatment within 7 days of enrolment, the median time from HIV diagnosis to cohort enrolment was 2 (IQR: 0–5) days. In multivariable analyses, no significant differences in VS were found between DTG/3TC and other first-line ART regimens among PWH initiating treatment with >100 000 copies/mL, and those who started within 7 days of enrolment or aged ≥50 years. Due to the low number of participants initiating DTG/3TC, we could not perform multivariable analyses in the rest of the subgroups analysed. The percentage of discontinuations of DTG/3TC due to AEs was <4% in all the subgroups analysed.

## Discussion

In this large multicentre cohort, we have shown high effectiveness and tolerability of DTG/3TC at 96 weeks among treatment-naive PWH. Moreover, we have shown that its effectiveness did not differ significantly to that of the most frequently prescribed three-drug regimens and has higher persistence and lower rate of discontinuations due to AEs than these regimens. To our knowledge, this is the first cohort study comparing long-term effectiveness and tolerability of DTG/3TC to other ARTs among treatment-naive PWH. These results are consistent with a previous analysis at 48 weeks of this same cohort.^12^

During the study period, the proportion of PWH who started ART with DTG/3TC increased gradually from 4.4% in 2018 to 24.8% in 2021. This trend reflects the changes in the national clinical guidelines for ART: regarding the recommendations for initial ART, DTG/3TC was not included as an option in 2018, it was considered alternative in 2019 and it was included as a preferred treatment since 2020.^14–16^

The assessment of long-term treatment effectiveness is especially important in real-life clinical practice, as the participants are usually not selected, have less strict follow-up procedures and might be more difficult to treat than those enrolled in clinical trials. However, there is very little evidence on DTG/3TC from long-term cohort studies, since the vast majority of them have assessed the outcomes at 48 weeks or less.^6^ Only two cohorts have analysed its long-term effectiveness in treatment-naive PWH. The REDOLA study enrolled 185 participants who started DTG/3TC in Spain, showing VS in 83.8% and 95.7% of the participants in the ITT and PP analysis, respectively, at 96 weeks.^10^ The URBAN cohort showed 77.8% effectiveness among 27 treatment-naive participants 3 years after starting DTG/3TC in Germany.^11^ Additionally, the TANDEM cohort enrolled 126 treatment-naive participants starting DTG/3TC in the USA who were followed for a median of 1.3 years (IQR: 0.8–1.8): this study reported that 83.3% of them remained virologically suppressed during follow-up but did not provide effectiveness data at specific timepoints.^17^ These were only descriptive studies, and neither of them compared DTG/3TC with other first-line treatments.

Our study provides the largest number of treatment-naive PWH starting DTG/3TC from a single cohort providing long-term outcomes. Also, since our cohort includes PWH starting different ARTs, we have been able to compare the effectiveness of DTG/3TC with other first-line three-drug regimens: this analysis is rarely reported in other cohorts. As the treatments were not randomized, these results are prone to confounding. Our estimates were adjusted for several potential confounders; however, as in all observational studies, we cannot exclude residual confounding. DTG/3TC was prescribed less frequently than other regimens to women, elderly persons or PWH with low education, low CD4 counts or high VLs. Our estimates were adjusted for these variables, among others, in the multivariable analysis.

Our findings are consistent with the 96-week analysis of the GEMINI studies, which showed non-inferiority of DTG + 3TC compared with DTG + TDF/FTC among treatment-naive PWH and high tolerability of DTG + 3TC.^8^ In these trials, there was a low proportion of VFs with no treatment-emergent resistance. We also found a low proportion of VFs in our participants who started DTG/3TC. Among those with available resistance tests, no resistance mutations developed at VF.

We found DTG/3TC to be highly effective in all the subgroups analysed. Among PWH with VL >100 000 copies/mL and those who started rapid treatment (within a week of their first visit), its effectiveness was comparable to other first-line regimens, even after adjusting for other risk factors. The low number of participants receiving DTG/3TC in the other subgroups (women and those with low CD4 counts or VL >500 000 copies/mL) does not allow us to draw firm conclusions. Spanish guidelines recommended not prescribing DTG/3TC as initial treatment to PWH with VLs >500 000 copies/mL during 2019 or <200 CD4 cells/μL throughout the study period.^14,15^ Also, due to the findings of the Tsepamo study,^18,19^ during 2019 and 2020 they also recommended not prescribing this regimen to women of childbearing age who were not using contraception.^14,15^

A high proportion of PWH were treated within the first week after cohort enrolment. Since PWH are enrolled in CoRIS at their first visit to the specialized clinical centre after HIV diagnosis, we can assume that they started treatment on a ‘test-and-treat’ basis and resistance test results were not available. Also, these participants were enrolled in the cohort very shortly after HIV diagnosis. The high effectiveness of DTG/3TC in this subgroup confirms the results of the STAT clinical trial.^2^ Other cohorts have shown high effectiveness of a ‘test-and-treat’ strategy with DTG/3TC in clinical practice^20–22^; however, neither the STAT trial nor these cohorts compared DTG/3TC with other treatment regimens.

We found DTG/3TC to have significantly higher persistence than all the other treatment regimens after adjusting for baseline clinical and demographic variables. Most of the other regimens’ main reason for discontinuation was simplification; however, these other regimens also had a higher risk of discontinuation due to AEs than DTG/3TC in the multivariable analysis. The accumulated incidence of treatment discontinuations increased gradually for all regimens except DTG + TDF/FTC, for which discontinuation rates were highest during the first 12 weeks of treatment: this two-pill regimen was probably prescribed awaiting resistance and HLA B5701 test results, and changed shortly after they were available.

There are several limitations of our study. Firstly, because it is an observational study, it could be prone to biases such as confounding by indication. In fact, PWH who started DTG/3TC had less risk factors for treatment failure than those receiving other regimens. However, our results were adjusted for these risk factors. Secondly, we could not compare our outcomes in some of the subgroups due to a low number of participants. The main strengths of our study are a national multicentre cohort that is representative of the newly diagnosed PWH in Spain^23^ and has strict quality control criteria; the reasonable high number of treatment-naive PWH, which provides the highest number treated with DTG/3TC from a single cohort published to date; and the comparative analysis with other first-line regimens.

### Conclusions

We have analysed the long-term effectiveness, persistence and tolerability of DTG/3TC among treatment-naive PWH in a large multicentre cohort. We found DTG/3TC to have comparable effectiveness to other widely used three-drug first-line regimens, in all the cohort and specifically among PWH with VL >100 000 copies/mL and those who started rapid treatment. Compared with all the first-line treatments analysed, DTG/3TC was the most durable and better tolerated regimen. These findings are consistent with the results of clinical trials and support the use of DTG/3TC as a first-line treatment for HIV infection.

## Supplementary Material

dkae456_Supplementary_Data

## Transparency declarations

I.S.-G. has received conference grants or speaker fees from ViiV Healthcare, Gilead, Merck Sharp & Dohme and Janssen. M.M. payment or honoraria for lectures, consulting fees or for attending meetings and/or travel from ViiV Healthcare, Janssen and Merck Sharp & Dohme. S.M. has been involved in speaking activities and has received grants for research from Gilead, Janssen Cilag, Merck Sharp & Dohme and ViiV Healthcare. I.J. has received teaching or advisory fees from ViiV Healthcare and Gilead. The remaining authors have no conflicts of interest to declare.

## Supplementary data

Tables S1–S4 are available as Supplementary data at *JAC* Online.
