# Supplementary material for: Long-term effectiveness and tolerability of dolutegravir/lamivudine in treatment-naive people with HIV: an analysis of a multicentre cohort at 96 weeks
Source: J Antimicrob Chemother. 2024 Dec 23;80(3):682–91. doi: 10.1093/jac/dkae456 (PMC11879157; doi:10.1093/jac/dkae456)
Supplement: dkae456_Supplementary_Data [file dkae456_supplementary_data.docx]

**SUPLEMENTARY TABLES**

**Table S1: Sociodemographic and clinical characteristics at ART initiation according to first-line antiretroviral regimen, CoRIS cohort, 2018-2023**

|  | **DTG/3TC** | **BIC/TAF/FTC** | **DTG/ABC/3TC** | **DRV/COBI/TAF/FTC** | **DTG+FTC/TDF** | **Total** | **p-value** |
| --- | --- | --- | --- | --- | --- | --- | --- |
|  | **N=472** | **N=1,134** | **N=273** | **N=180** | **N=300** | **N=2,359** |  |
| Sex [N (%)] |  |  |  |  |  |  | 0.035 |
| Male | 438 (92.8) | 1,007 (88.8) | 247 (90.5) | 155 (86.1) | 261 (87.0) | 2,108 (89.4) |  |
| Female | 34 (7.2) | 127 (11.2) | 26 (9.5) | 25 (13.9) | 39 (13.0) | 251 (10.6) |  |
| Age, years [N (%)] |  |  |  |  |  |  | 0.006 |
| <30 | 178 (37.7) | 330 (29.1) | 72 (26.4) | 60 (33.3) | 95 (31.7) | 735 (31.2) |  |
| 30-49 | 239 (50.6) | 627 (55.3) | 162 (59.3) | 93 (51.7) | 148 (49.3) | 1,269 (53.8) |  |
| ≥50 | 55 (11.7) | 177 (15.6) | 39 (14.3) | 27 (15.0) | 57 (19.0) | 355 (15.0) |  |
| Median (IQR) | 33.0 (26.8; 41.9) | 36.1 (29.0; 45.1) | 36.6 (29.4; 45.2) | 35.8 (28.0; 42.4) | 35.4 (27.7; 46.2) | 35.6 (28.2; 44.5) | <0.001 |
| Transmission category [N (%)] |  |  |  |  |  |  | 0.001 |
| Sex between men | 378 (80.1) | 800 (70.5) | 188 (68.9) | 119 (66.1) | 206 (68.7) | 1,691 (71.7) |  |
| Sex between men and women | 76 (16.1) | 278 (24.5) | 62 (22.7) | 48 (26.7) | 70 (23.3) | 534 (22.6) |  |
| Other | 9 (1.9) | 17 (1.5) | 6 (2.2) | 6 (3.3) | 8 (2.7) | 46 (1.9) |  |
| Unknown | 9 (1.9) | 39 (3.4) | 17 (6.2) | 7 (3.9) | 16 (5.3) | 88 (3.7) |  |
| Educational level [N (%)] |  |  |  |  |  |  | <0.001 |
| No education/compulsory education | 90 (19.1) | 227 (20.0) | 64 (23.4) | 49 (27.2) | 61 (20.3) | 491 (20.8) |  |
| Secondary/university education | 326 (69.1) | 651 (57.4) | 142 (52.0) | 80 (44.4) | 148 (49.3) | 1,347 (57.1) |  |
| Other | 5 (1.1) | 12 (1.1) | 5 (1.8) | 5 (2.8) | 0 (0.0) | 27 (1.1) |  |
| Unknown | 51 (10.8) | 244 (21.5) | 62 (22.7) | 46 (25.6) | 91 (30.3) | 494 (20.9) |  |
| Country of origin [N (%)] |  |  |  |  |  |  | 0.012 |
| Spain | 231 (48.9) | 522 (46.0) | 127 (46.5) | 91 (50.6) | 172 (57.3) | 1,143 (48.5) |  |
| No Spain | 240 (50.8) | 603 (53.2) | 146 (53.5) | 89 (49.4) | 128 (42.7) | 1,206 (51.1) |  |
| Unknown | 1 (0.2) | 9 (0.8) | 0 (0.0) | 0 (0.0) | 0 (0.0) | 10 (0.4) |  |
| Viral load, copies/mL [N (%)] |  |  |  |  |  |  | <0.001 |
| ≤100,000 | 328 (69.5) | 541 (47.7) | 145 (53.1) | 100 (55.6) | 101 (33.7) | 1,215 (51.5) |  |
| >100,000 | 122 (25.8) | 495 (43.7) | 110 (40.3) | 67 (37.2) | 185 (61.7) | 979 (41.5) |  |
| Unknown | 22 (4.7) | 98 (8.6) | 18 (6.6) | 13 (7.2) | 14 (4.7) | 165 (7.0) |  |
| Median (IQR) Log VL | 4.6 (3.9; 5.1) | 5.0 (4.3; 5.6) | 4.9 (4.2; 5.5) | 4.8 (4.2; 5.5) | 5.3 (4.7; 5.8) | 4.9 (4.2; 5.5) | <0.001 |
| CD4 cell count, cells/microL [N (%)] |  |  |  |  |  |  |  |
| <200 | 17 (3.6) | 271 (23.9) | 44 (16.1) | 45 (25.0) | 81 (27.0) | 458 (19.4) | <0.001 |
| 200-500 | 238 (50.4) | 469 (41.4) | 118 (43.2) | 68 (37.8) | 134 (44.7) | 1,027 (43.5) |  |
| >=500 | 190 (40.3) | 273 (24.1) | 90 (33.0) | 53 (29.4) | 63 (21.0) | 669 (28.4) |  |
| Unknown | 27 (5.7) | 121 (10.7) | 21 (7.7) | 14 (7.8) | 22 (7.3) | 205 (8.7) |  |
| Median (IQR) | 458 (340; 652) | 347 (188; 518) | 411 (235; 589) | 369 (179; 563) | 323 (176; 483) | 382 (230; 548) | <0.001 |
| Hepatitis C virus antibodies [N (%)] | |  |  |  |  |  | <0.001 |
| Negative | 413 (87.5) | 868 (76.5) | 213 (78.0) | 136 (75.6) | 256 (85.3) | 1,886 (79.9) |  |
| Positive | 11 (2.3) | 31 (2.7) | 14 (5.1) | 6 (3.3) | 11 (3.7) | 73 (3.1) |  |
| Unknown | 48 (10.2) | 235 (20.7) | 46 (16.8) | 38 (21.1) | 33 (11.0) | 400 (17.0) |  |
| Hepatitis B virus surface antigen [N (%)] | |  |  |  |  |  | 0.030 |
| Negative | 343 (72.7) | 815 (71.9) | 202 (74.0) | 114 (63.3) | 210 (70.0) | 1,684 (71.4) |  |
| Positive | 1 (0.2) | 20 (1.8) | 2 (0.7) | 6 (3.3) | 4 (1.3) | 33 (1.4) |  |
| Unknown | 128 (27.1) | 299 (26.4) | 69 (25.3) | 60 (33.3) | 86 (28.7) | 642 (27.2) |  |
| Previous AIDS diagnosis [N (%)] |  |  |  |  |  |  | <0.001 |
| No | 462 (97.9) | 1035 (91.3) | 242 (88.6) | 161 (89.4) | 261 (87.0) | 2,161 (91.6) |  |
| Yes | 10 (2.1) | 99 (8.7) | 31 (11.4) | 19 (10.6) | 39 (13.0) | 198 (8.4) |  |

IQR, interquartile range; DTG/3TC, dolutegravir/lamivudine; BIC/FTC/TAF, bictegravir/emtricitabine/tenofovir alafenamide; DTG/3TC/ABC, dolutegravir/lamivudine/abacavir; DRV/COBI/FTC/TAF, darunavir/cobicistat/emtricitabine/tenofovir alafenamide; DTG+FTC/TDF, dolutegravir+emtricitabine/tenofovir disoproxil fumarate.

**Table S2: Selection of the study population for the ITT and OT analyses of viral suppression, immunological response and virologic failure at 96 weeks from ART initiation according to first-line antiretroviral regimen, CoRIS cohort, 2018-2023**

|  | **DTG/3TC** | **BIC/FTC/TAF** | **DTG/3TC/ABC** | **DRV/COBI/**  **FTC/TAF** | **DTG+FTC/TDF** | **Total** |
| --- | --- | --- | --- | --- | --- | --- |
|  | **N=472** | **N=1,134** | **N=273** | **N=180** | **N=300** | **N=2,359** |
| **Overall exclusions** |  |  |  |  |  |  |
| Death before the assessment window | 3 (0.6%) | 12 (1.1%) | 6 (2.2%) | 0 (0.0%) | 4 (1.3%) | 25 (1.1%) |
| Last visit to the centre before the assessment window | 58 (12.3%) | 125 (11.0%) | 31 (11.4%) | 19 (10.6%) | 25 (8.3%) | 258 (10.9%) |
| No visit in the assessment window | 35 (7.4%) | 72 (6.3%) | 19 (7.0%) | 17 (9.4%) | 27 (9.0%) | 170 (7.2%) |
|  |  |  |  |  |  |  |
| **Exclusions for Viral suppression analyses** |  |  |  |  |  |  |
| Available visit in the assessment window but missing viral load | 27 (5.7%) | 44 (3.9%) | 10 (3.7%) | 9 (5.0%) | 18 (6.0%) | 108 (4.6%) |
| **ITT analyses** | **349 (73.9%)** | **881 (77.7%)** | **207 (75.8%)** | **135 (75.0%)** | **226 (75.3%)** | **1,798 (76.2%)** |
| Treatment discontinuation before 96 weeks* | 31 (8.9%) | 110 (12.5%) | 62 (30.0%) | 44 (32.6%) | 167 (73.9%) | 414 (23.0%) |
| **OT analyses** | **318 (67.4%)** | **771 (68.0%)** | **145 (53.1%)** | **91 (50.6%)** | **59 (19.7%)** | **1,384 (58.7%)** |
|  |  |  |  |  |  |  |
| **Exclusions for Immunological recovery analyses** |  |  |  |  |  |  |
| Available visit in the assessment window but missing CD4 | 31 (6.6%) | 128 (11.3%) | 19 (7.0%) | 20 (11.1%) | 21 (7.0%) | 219 (9.3%) |
| **ITT analyses** | **345 (73.1%)** | **797 (70.3%)** | **198 (72.5%)** | **124 (68.9%)** | **223 (74.3%)** | **1,687 (71.5%)** |
| Treatment discontinuation before 96 weeks* | 33 (9.6%) | 105 (13.2%) | 63 (31.8%) | 38 (30.6%) | 168 (75.3%) | 407 (24.1%) |
| **OT analyses** | **312 (66.1%)** | **692 (61.0%)** | **135 (49.5%)** | **86 (47.8%)** | **55 (18.3%)** | **1,280 (54.3%)** |
|  |  |  |  |  |  |  |
| **Exclusions for Virological Failure Analyses** |  |  |  |  |  |  |
| Not achieving Viral suppression | 34 (7.2%) | 82 (7.2%) | 15 (5.5%) | 24 (13.3%) | 28 (9.3%) | 183 (7.8%) |
| Not viral load available after Viral suppression | 59 (12.5%) | 101 (8.9%) | 26 (9.5%) | 20 (11.1%) | 34 (11.3%) | 240 (10.2%) |
| **ITT analyses** | **386 (81.8%)** | **972 (85.7%)** | **235 (86.1%)** | **141 (78.3%)** | **241 (80.3%)** | **1,975 (83.7%)** |
| Treatment discontinuation before 96 weeks* | 32 (8.3%) | 120 (12.3%) | 72 (30.6%) | 50 (35.5%) | 177 (73.4%) | 451 (22.8%) |
| **OT analyses** | **354 (75.0%)** | **852 (75.1%)** | **163 (59.7%)** | **91 (50.6%)** | **64 (21.3%)** | **1,524 (64.6%)** |

ITT, intention-to-treat; OT, on-treatment; DTG/3TC, dolutegravir/lamivudine; BIC/FTC/TAF, bictegravir/emtricitabine/tenofovir alafenamide; DTG/3TC/ABC, dolutegravir/lamivudine/abacavir; DRV/COBI/FTC/TAF, darunavir/cobicistat/emtricitabine/tenofovir alafenamide; DTG+FTC/TDF, dolutegravir+emtricitabine/tenofovir disoproxil fumarate.

All percentages are calculated over the total number of individuals in each group, except those for ^*^Treatment discontinuation before 96 weeks that are calculated over the total number of individuals included in the ITT analyses

**Tables S3A and S3B: Detailed description of virologic failures after viral suppression among treatment-naïve individuals starting with DTG/3TC, CoRIS cohort, 2018-2023**

| Case | Age at VF | Sex | Baseline CD4 (cells/microL) | Baseline viral load | Time to VF since start of treatment (weeks) | Treatment at VF | Time with last treatment (weeks) | Viral load at VF (copies/ml) | Treatment after VF | Outcome |
| --- | --- | --- | --- | --- | --- | --- | --- | --- | --- | --- |
| 1 | 31 | Male | 15 | 353 898 | 42.4 | DTG/3TC | 42.4 | 1179 | DTG/3TC | Viral suppression |
| 2 | 36 | Male | 377 | 2 000 | 26.9 | DTG/3TC | 26.9 | 3700 | DTG/3TC | No follow-up |
| 3 | 29 | Male | 429 | 219 | 22.6 | Without ART | 3.6 | 17800 | DTG/3TC | Viral suppression |
| 4 | 29 | Male | 152 | 3 390 | 89.0 | Without ART | 20.0 | 7794 | DTG/3TC | No follow-up |
| 5 | 41 | Male | 46 | 3 090 000 | 73.1 | DTG/3TC | 73.1 | 128 | DTG/3TC | No viral suppression* |
| 6 | 47 | Male | 469 | 271 000 | 114.9 | ABC/DTG/3TC | 56 | 73 | ABC/DTG/3TC | Viral suppression |

| Case | Resistance at baseline | Resistance at VF |
| --- | --- | --- |
| 1 | Reverse transcriptase inhibitors: no resistance  Integrase inhibitors: not available | Reverse transcriptase inhibitors: could not be amplified  Integrase inhibitors: no resistance |
| 2 | Not available | Not available |
| 3 | Reverse transcriptase inhibitors: no resistance  Integrase inhibitors: no resistance | Not available |
| 4 | Reverse transcriptase inhibitors: no resistance  Integrase inhibitors: not available | Reverse transcriptase inhibitors: no resistance  Integrase inhibitors: no resistance |
| 5 | Reverse transcriptase inhibitors: no resistance  Integrase inhibitors: no resistance | Reverse transcriptase inhibitors: no resistance  Integrase inhibitors: no resistance |
| 6 | Reverse transcriptase inhibitors: V179D  Integrase inhibitors: not available | Reverse transcriptase inhibitors: V179D  Integrase inhibitors: no resistance |

ART: antiretroviral therapy. VF: virologic failure

*Viral load 251 copies/ml two months after VF. No further viral load measurements available due to administrative censoring of the database. The patient had suboptimal adherence to treatment according to the treating clinician.

**Table S4: Viral suppression and treatment discontinuation due to adverse events at 96 weeks from ART initiation according to first-line antiretroviral regimen, in specific subgroups, CoRIS cohort, 2018-2023**

|  | **Viral Suppression** | | **Treatment discontinuations due to AE [N/N with data (%)]** |
| --- | --- | --- | --- |
|  | **N/N with data (%)** | **Adjusted OR (95% CI)*** |  |
| **ART initiation with CD4 cell count < 200 cells/μL** | | |  |
| DTG/3TC | 10/12 (83.3) |  | 0/17 (0.0) |
| BIC/FTC/TAF | 177/214 (82.7) |  | 9/271 (3.3) |
| DTG/3TC/ABC | 31/35 (88.6) |  | 2/44 (4.5) |
| DRV/COBI/FTC/TAF | 30/39 (76.9) |  | 4/45 (8.9) |
| DTG+FTC/TDF | 55/66 (83.3) |  | 15/81 (18.5) |
|  | | |  |
| **ART initiation with HIV RNA VL > 100,000 copies/ml** | | |  |
| DTG/3TC | 84/97 (86.6) | Ref. | 2/122 (1.6) |
| BIC/FTC/TAF | 334/386 (86.5) | 1.37 (0.66; 2.85) | 12/495 (2.4) |
| DTG/3TC/ABC | 76/85 (89.4) | 1.67 (0.68; 4.10) | 8/110 (7.3) |
| DRV/COBI/FTC/TAF | 41/51 (80.4) | 1.14 (0.45; 2.91) | 5/67 (7.5) |
| DTG+FTC/TDF | 123/143 (86.0) | 1.48 (0.60; 3.65) | 24/185 (13.0) |
|  | | |  |
| **ART initiation with HIV RNA VL > 500,000 copies/ml** | | |  |
| DTG/3TC | 22/25 (88.0) |  | 0/37 (0.0) |
| BIC/FTC/TAF | 145/169 (85.8) |  | 7/206 (3.4) |
| DTG/3TC/ABC | 34/35 (97.1) |  | 0/44 (0.0) |
| DRV/COBI/FTC/TAF | 20/24 (83.3) |  | 1/30 (3.3) |
| DTG+FTC/TDF | 57/66 (86.4) |  | 12/86 (14.0) |
|  | | |  |
| **ART initiation within 7 days of enrolment** | | |  |
| DTG/3TC | 177/191 (92.7) | Ref. | 3/258 (1.2) |
| BIC/FTC/TAF | 510/565 (90.3) | 1.27 (0.67; 2.41) | 18/709 (2.5) |
| DTG/3TC/ABC | 64/69 (92.8) | 2.06 (0.55; 7.69) | 4/90 (4.4) |
| DRV/COBI/FTC/TAF | 54/62 (87.1) | 1.06 (0.41; 2.71) | 6/85 (7.1) |
| DTG+FTC/TDF | 139/154 (90.3) | 1.44 (0.73; 2.84) | 23/194 (11.9) |
|  |  |  |  |
| **Women** |  |  |  |
| DTG/3TC | 27/27 (100.0) |  | 1/34 (2.9) |
| BIC/FTC/TAF | 98/106 (92.5) |  | 8/127 (6.3) |
| DTG/3TC/ABC | 15/17 (88.2) |  | 2/26 (7.7) |
| DRV/COBI/FTC/TAF | 14/18 (77.8) |  | 1/25 (4.0) |
| DTG+FTC/TDF | 30/35 (85.7) |  | 4/39 (10.3) |
|  | |  |  |
| **Age at ART initiation ≥ 50 years** | |  |  |
| DTG/3TC | 39/43 (90.7) | Ref. | 2/55 (3.6) |
| BIC/FTC/TAF | 116/131 (88.5) | 1.49 (0.32; 7.04) | 4/177 (2.3) |
| DTG/3TC/ABC | 25/29 (86.2) | 0.74 (0.17; 3.29) | 1/39 (2.6) |
| DRV/COBI/FTC/TAF | 15/19 (78.9) | 0.71 (0.12; 4.14) | 3/27 (11.1) |
| DTG+FTC/TDF | 45/47 (95.7) | 5.06 (1.01; 25.31) | 11/57 (19.3) |

ART, antiretroviral treatment; OR, odds ratio; CI, confidence interval; AE, adverse evento; DTG/3TC, dolutegravir/lamivudine; BIC/FTC/TAF, bictegravir/emtricitabine/tenofovir alafenamide; DTG/3TC/ABC, dolutegravir/lamivudine/abacavir; DRV/COBI/FTC/TAF, darunavir/cobicistat/emtricitabine/tenofovir alafenamide; DTG+FTC/TDF, dolutegravir+emtricitabine/tenofovir disoproxil fumarate.

*Adjusted for sex, age at ART initiation, transmission category, educational level, country of origin, CD4 cell count and viral load within 6 months previous to ART initiation, presence of hepatitis C virus antibodies, presence of hepatitis B virus surface antigen, and previous AIDS diagnosis at ART initiation.
